# Polyborosiloxanes (PBS): Evolution of Approaches to the Synthesis and the Prospects of Their Application

**DOI:** 10.3390/polym14224824

**Published:** 2022-11-09

**Authors:** Fedor V. Drozdov, Elizaveta A. Manokhina, Tran D. Vu, Aziz M. Muzafarov

**Affiliations:** 1 A. N. Nesmeyanov Institute of Organoelement Compounds, Russian Academy of Sciences, 119991 Moscow, Russia; 2 Enikolopov Institute of Synthetic Polymeric Materials, Russian Academy of Sciences, 117393 Moscow, Russia; 3 Institute of Tropical Durability, Joint Russia-Vietnam Tropical Science and Technology, Hanoi 122103, Vietnam

**Keywords:** borosiloxanes, siloxanes, non-covalent interactions, tixotropic fluids, SiBOC

## Abstract

The mini-review deals with borosiloxanes as a class of organoelement compounds that comprise Si-O-B bonds, including individual compounds and polymeric structures. The borosiloxanes first synthesized in the 1950s using simple methods demonstrated very unusual properties but were hydrolytically unstable. However, in recent times, synthetic methods have changed significantly, which made it possible to synthesize borosiloxanes that are resistant to external factors, including atmospheric moisture. Borosiloxanes became important due to their unique properties. For example, borosiloxane liquids acquire a thixotropic behavior due to donor-acceptor interchain interactions. In addition, borosiloxanes are used to produce flame-retardant ceramics. An analysis of the literature sources shows that no review has yet been completed on the topic of borosiloxanes. Therefore, we decided that even a brief outlook of this area would be useful for researchers in this and related fields. Thus, the review shows the evolution of the synthesis methods and covers the studies on the properties of these unique molecules, the latest achievements in this field, and the prospects for their application.

## 1. Classification of PBS

Currently, a wide range of compounds are referred to as polyborosiloxanes. Therefore, let us define this term at once in order to avoid confusion. Initially, the name “borosiloxanes” was given to individual compounds comprising Si-O-B bonds. Various derivatives of boric and aryl(alkyl)boric acids or boron halides, such as trimethylsilyl borates and mixed borosiloxane cycles that will be discussed below, may be regarded as such compounds. Therefore, borosiloxanes can be thought of as inorganic derivatives of boric acid with an organosilicon framing. Later, the “PBS” term was accepted that already indicated the polymeric structure of these compounds. In terms of structure, PBSs also contain Si-O-B bonds, but these compounds are oligomeric or polymeric, rather than individual compounds. PBSs are synthesized in a similar way, but the functionality of the starting monomers is larger than two, which makes it possible to obtain polymers with diverse structures. It should be mentioned here that the sol-gel methods of PBS synthesis became especially important because of their simplicity and efficiency, as well as availability of the starting monomers. The Si-O-B bond in PBS is usually hydrolytically unstable, which imposes a number of restrictions on their use, although, as we will show below, methods for synthesizing PBS are being improved, which allows their stability to be increased considerably.

The second generation of PBS includes organic derivatives of substituted boric acids comprising no Si-O-B bonds as such, which means that they are hydrolytically stable. Based on the combination of their properties, they can also be classified as PBSs. The PBSs of this kind usually have the ≡Si-Ar-B(OH)_2_ functionality. The arylborate residue is more stable than, for example, the alkylborate one. The aryl moiety can be represented either directly by a substituted 1,4-phenylene or by aromatic groups with spacers, depending on the method for the synthesis of the respective PBSs. Yet, another important type of PBS should certainly be mentioned, namely, poly(carborane siloxanes) [1,2]. The carborane framework in compounds of this class is linked directly or through a spacer to the polydimethylsiloxane part. These compounds have a number of interesting and important properties, such as high thermal resistance along with high thermooxidative and chemical stability, which determined their wide distribution under the DEXSIL trade mark [3]. However, the properties of poly(carborane siloxanes) are different from those of the “classical” PBSs that are mainly determined by the donor-acceptor nature of interchain interactions. Therefore, polycarboranesiloxanes are not considered in this review. If you are interested in their chemistry and applications, please see other reviews [4,5].

## 2. Application Fields of PBS with Inorganic Moieties

Historically, the interest in polyborosiloxanes arose because of the unusual unique properties of the so-called “polyborosiloxane fluids”. The first references to the synthesis and study of the properties of such molecules, which are oligomers with alternating Si-O-B moieties, were given in conference abstracts of Soviet scientists in the 1950–1960s and were cited in a number of reviews and books [6,7,8].

**Figure 1:**
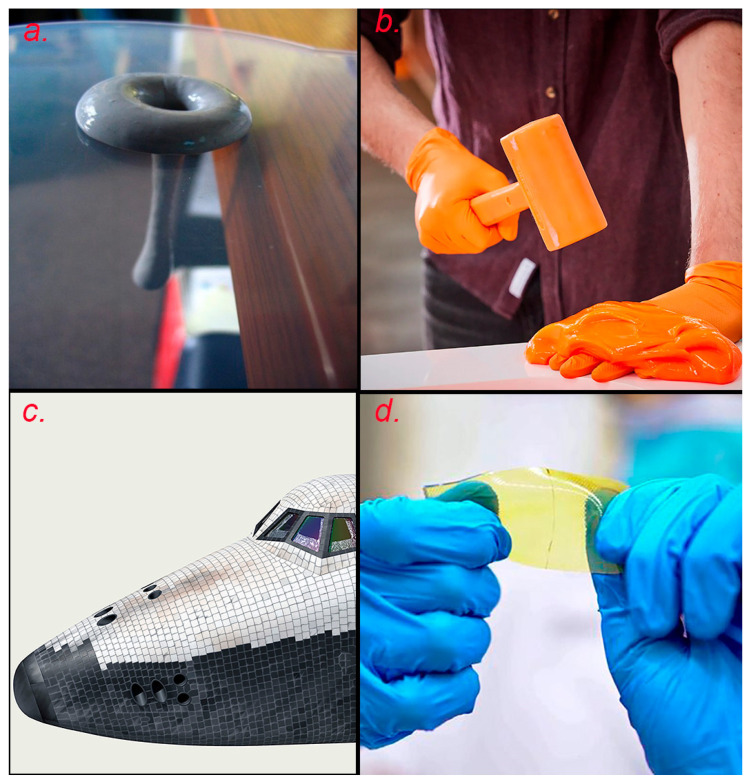
Representation of the use of PBS as rheological liquids (**a**) for the manufacture of protection against mechanical damage (**b**), as fire-resistant ceramics (**c**) and in self-healing materials (**d**).

**Figure 2:**
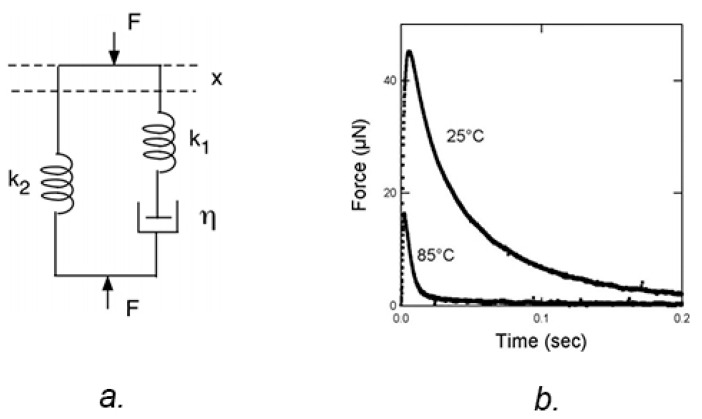
Rheological model of Silly Putty (**a**); dependence of relaxation on time (**b**).

In these works, the effect of the monomer ratio on the molecular weight characteristics, the viscosity of the oligomers obtained, and their stability was studied in detail. Moreover, more complex oligomeric structures comprising other heteroatoms were also studied [9]. In later years, beginning in the 1970s, they became popular due to the “liquid plasticine” or “hand gum” toys for children created with these compounds (Figure 1a). Names such as “bouncing putty” or “Silly Putty” can be found in the English-language literature. The interest in these materials waxed and waned now and again. At an early stage of studies on polyborsiloxanes, masters of silicon chemistry such as Andrianov and Voronkov provided their interpretation of the observed effects [10,11,12]. However, detailed studies on the rheological properties of these systems have been performed only in the current century. Liquids of this kind can possess plastic and elastic properties simultaneously but behave as Newtonian fluids [13,14]. Unlike siloxane fluids that typically do not have such a rheological behavior, in polyborsiloxane fluids it is determined by a combination of high flexibility of siloxane chains, on the one hand, and the formation of donor-acceptor interchain bonds between the vacant orbitals of boron atoms with unshared electron pairs of the oxygen atoms, on the other hand. As a result of this interaction between the neighboring chains of PBSs, a dynamic network is formed, which determines the physical properties of the material based on them [15]. In some works, the physico-mechanical and rheological properties of silly putty were studied and models were built based on approximations of the data obtained. For example, a parallel model containing a Maxwell element coupled with an elastic element was suggested (Figure 2a) [14].

It was shown in [16] how the relaxation of silly putty varied with time depending on the temperature (Figure 2b). It is evident from the plots obtained that the sample relaxation occurs faster at higher temperatures, which is obviously associated with the predominance of flow and rearrangement of noncovalent interactions. Thus, a change in the number, nature, and functionality of organoborate moieties, along with the length and functionality of the siloxane chains, results in strong changes in the aggregate state, physico-mechanical, and rheological properties of such PBS [17].

**Figure 3:**
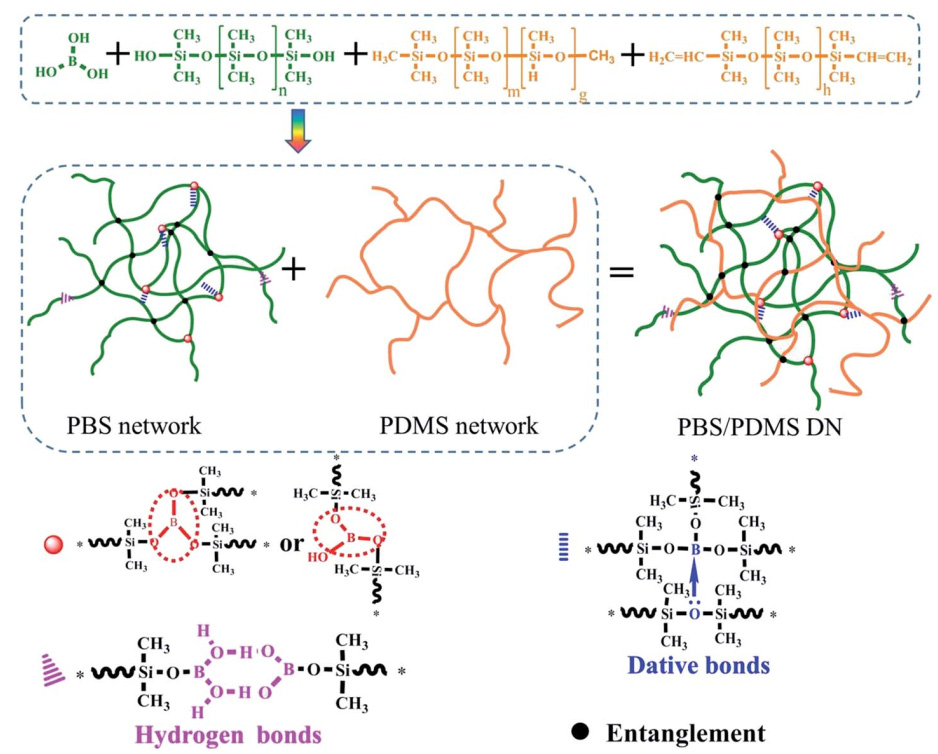
Formation of interpenetrating meshes of cross-linked siloxanes and siloxanes with terminal silanol groups with boric acid. Reprinted with permission from [28]. Copyright 2022 Royal Society of Chemistry.

The feasibility of changing the physical properties of polyborosiloxane fluids in a wide range makes it possible to use such PBS in many applications, e.g., as adhesives, sealants, polymer coatings, and structured liquids. One of the examples where PBS-based rheological fluids are used is organoborate curing with polysiloxanes, which makes it possible to obtain materials with various elastic-viscosity characteristics, from gels to rubbers [18,19,20]. The combination of viscoelastic properties of rheological fluids based on PBSs opens up the possibility of their use as damping fluids and, in particular, in shock protection materials. When such a material undergoes a shock, it behaves as an elastic Hookean body dissipating the shock energy into heat. However, viscoelastic rheological fluids can flow, unlike various polymeric analogues that crack after a mechanical impact, and, due to the restructuring of the labile non-covalent bonds, the former exhibits a self-healing effect, which allows protective materials based on them to be used repeatedly (Figure 1b). Vast patent literature deals with the use of PBSs as components of protective suits, equipment, and motorcycle and bicycle protection. As an example, PBSs are used by Dow Corning in formulations with ethylenevinylacetate to create protection for football players [21] and light protective motocross suits [22]. Other examples include the development of a foam (“J-Foam”) by Design Blue company based on a formulation of a PBS gel and dry polyurethane for mountain skiing protection [23] and a shock-proof coating based on PBS and Kevlar [24]. It was shown that the joint curing of methylvinyl rubber and PBSs with benzoyl peroxide created rubbers with enhanced damping properties, tan δ > 0.3 in the range from −25 to 125 °C, due to the formation of additional noncovalent interactions that efficiently transmit and dampen the energy of an external impact [25]. Moreover, a resin based on vinyl triethoxysilane and boric acid showed a higher thermal resistance and, after heat treatment, it had a higher compressive strength than conventional silicone. Dense layers of the SiBOC composite are formed after annealing [26]. It is worthy of note that borosiloxanes can be obtained by the mechanoactivation of, for example, phenylsilsesquioxanes and boric acid. In this case, polymer structures consisting of mixed cycles are formed [27]. Self-healing conductive composites have aroused huge interest in recent years because of their important applications, especially as self-repairing electrodes in stretchable and reconfigurable electronics. Such composites are built as interpenetrating meshes of cross-linked siloxanes and siloxanes with terminal silanol groups with boric acid (Figure 3). Due to covalent bonds, PDMSs provide strength and elasticity, while PBSs enable self-healing due to labile Si-O-B bonds [28].

Works dealing with the production of silicon carbide fibers from polyborodiphenylsiloxanes are available in [29]. Data on the use of PBSs as anaerobic adhesives also exist [30].

Similar composites consisting of interpenetrating PBSs and polyurethane meshes were developed for creating highly flexible, recyclable, self-healing materials for a health monitoring sensor [31]. Similar formulations were developed to imitate human skin [32,33]. Composites based on PBSs filled with magnetic nanoparticles were created and their mechano-rheological behavior was studied. Composites of this kind can find use in aircrafts, in mechanical engineering, and as damping fluids [34,35].

PBSs have high thermal resistance as reported in many works [36,37], so they are actively used as starting compounds for borosiloxane ceramics (SiBOC) [38,39], and borosiloxane oxynitride ceramic materials (SiBON) [40]. Borosiloxane ceramics are obtained using the sol-gel method from boric acid and polysiloxanes. Polyborosiloxane sols have a remarkable spinnability where boron atoms are homogeneously incorporated into the linear Si-O-Si skeleton through Si-O-B bridges. SiBOC fibers with a diameter of 10 μm have been shown to withstand temperatures up to 1500 °C and can be used, for example, for cladding spacecrafts (Figure 1c) [41,42]. SiBON ceramics can be obtained by thermal nitriding polyborosiloxane or by the pyrolysis of polyborosilazane in anhydrous ammonia. Recently, SiBON fibers were obtained using this method from polyborosilazane [43,44].

The combination of the good rheological properties of PBSs and high thermal stability allow for cross-linked PBSs to be used as flame retardants. For example, a layered double hydroxide (LDH)-based composite with a silicone coating applied in situ using PBSs was obtained [45]. These composites showed high thermal stability up to 700 °C with low mass losses. Moreover, amino-terminated phosphorous polyborosiloxanes (N-PBSi) also exhibiting flame retardant properties were obtained [46].

Forming reversibly destructible covalent interchain bonds, PBSs have recently been actively used to create self-healing soft coatings (Figure 1d), which will be discussed in Section 4.

The interest in PBSs is currently growing in other areas, for example, their applications in soft robots [47] and in lithium ion batteries [48] were mentioned. A PBS was used to create ion-selective electrodes for fluoride anions [36]. PBSs were even used as a “migrating earplug” in the treatment of the tympanic cavity [49]. Thermally conductive elastomers based on PBSs and liquid metal microdroplets with a 401% fracture strain and 2164 J/m^2^ fracture resistance were obtained [50].

Thus, analysis of the currently available literature on the properties of polyborosiloxanes allows us to conclude that these compounds have a number of unique properties that enable them to be applicable in innovative fields of modern technologies. The two most outstanding properties of PBSs include the ability to form labile noncovalent interchain bonds and their high thermal stability. The first property opens prospects for the use of PBSs as rheological fluids, while the second one makes them potentially useful as precursors for the production of flame retardant ceramics and fibers. However, as mentioned above, the potential of PBSs is not limited to these two areas of application.

## 3. Methods for Synthesizing PBS with Si-O-B Inorganic Moieties

**Scheme 1:**
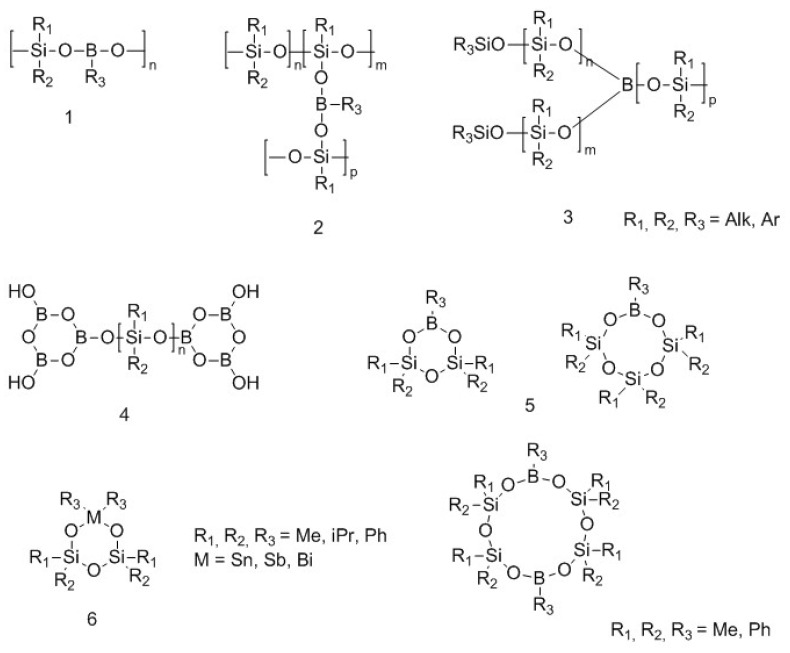
Various borosiloxane and polyborsiloxane derivatives.

Polyborosiloxanes (PBS) constitute a broad class of organoelement compounds containing Si-O-B bonds (Scheme 1). Their molecules can have the following structures: linear polymers with a regular Si-O-B structure 1, polysiloxanes cross-linked with organoborate moieties 2, branched polymers 3, polymers modified with boric acid and its esters 4, cycloborosiloxane with structure 5, and mixed cyclometalloborosiloxanes 6.

**Scheme 2:**
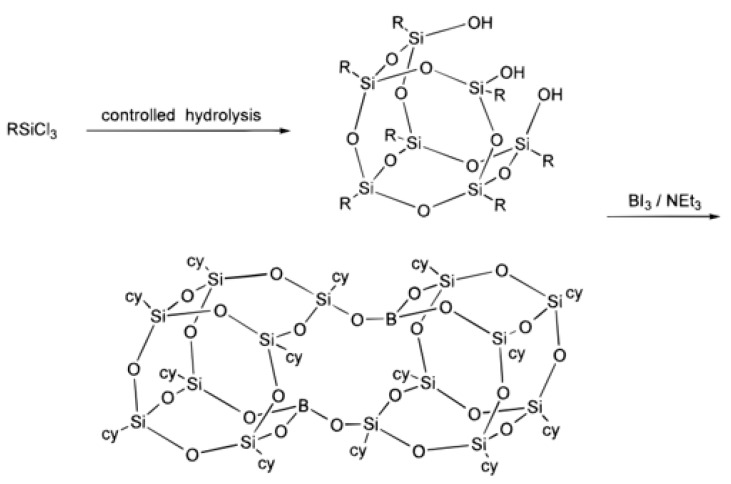
Synthesis of a polyhedral silsesquioxane (POSS) with borate bridges.

It is interesting to note that more complex structures can also be formed, depending on the functionality and ratio of the starting reagents. For example, the reactions of RSi(OH)_3_ (R = t-Bu or cyclo-C_6_H_11_) and boronic acid RB(OH)_2_ in a 2:3 molar ratio in toluene provide tricyclic structures with vertices represented by silicon atoms [51]. Mixed rings were obtained by the reaction of coordinated oxides and carbonates of antimony, tin, and bismuth with boroxines R_3_B_3_O_3_ (R = Me or OMe) [52]. Moreover, other methods for the synthesis of borocyclosiloxanes and their organoelement analogues were also studied [53]. It was found that mixed borosiloxane rings of type 5 are readily rearranged into each other at an elevated temperature in the presence of acid or base catalysts. It is interesting to note that the rearrangements result in larger rings mainly containing one boron atom. The same results were obtained in attempts to perform the polymerization with a ring opening under conditions typical of cyclosiloxanes. Borocyclosiloxanes with phenyl substituents 5 (R_1_, R_2_, R_3_ = Ph) crystallize and their structures were determined using single-crystal X-ray diffraction analysis [54,55]. Borocyclosiloxanes are stable compounds under ordinary conditions. Their stability increases on the replacement of alkyl substituents with more strongly branched ones or with phenyls. Apart from borocyclosiloxanes, polyhedral silsesquioxanes (PSS) with borate bridges were also obtained (Scheme 2). Structures of this type were obtained from an incompletely condensed trisilanol using a treatment with boron triiodide [56].

The structure of the product was confirmed using NMR spectroscopy and single-crystal X-ray diffraction analysis. Surprisingly, this product was stable against Lewis acids: OEt_2_, THF, NMe_3_O, and PPh_3_O and against strong nucleophiles, such as alkali metals, alkoxides, and siloxanolates that usually cause the cleavage and rearrangements of siloxane bonds.

Thus, it may be concluded that borosiloxanes with individual structures strongly differ in chemical properties. For example, trimethylsilyl borate is an extremely unstable compound that can be stored only in sealed tubes in an inert medium. On the other hand, borocyclosiloxanes are hydrolytically stable, while POSS with borate moieties are stable even against strong nucleophiles.

**Scheme 3:**
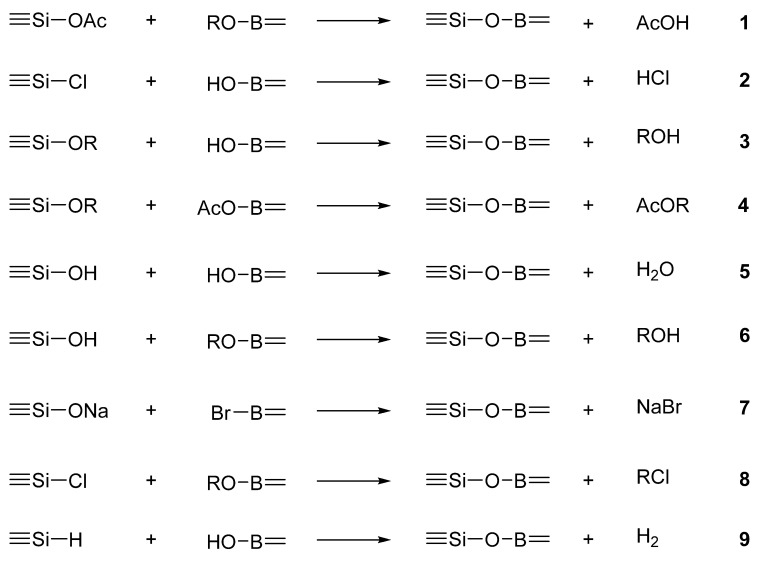
Various methods to form the Si-O-B bond.

As noted previously, the development of polyborosiloxane chemistry began in the 1950s when several scientific teams headed by Andrianov and Voronkov [6,7,8] had succeeded in synthesizing compounds with Si-O-B bonds from available boron derivatives such as boric acid (Scheme 3, reactions 2, 3, 5, 9), alkylborates (Scheme 3, reactions 1, 6, 8) and acetoxyborates (Scheme 3, reaction 4), haloborates (Scheme 3, reaction 7) with various silicon derivatives using conventional sol-gel condensation methods [15,57,58].

**Scheme 4:**
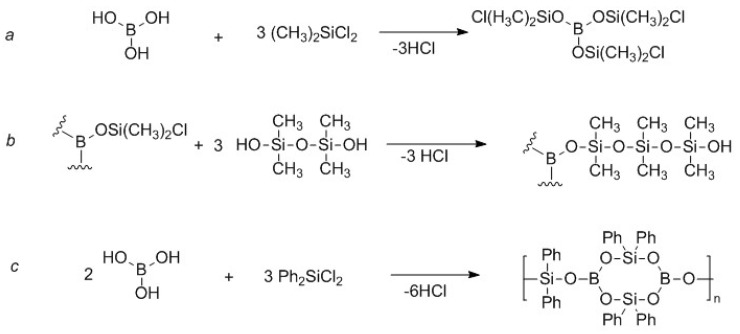
Directed synthesis of PBS with various structures: synthesis of trifunctional borosiloxane with chlorosilyl groups (**a**); its reaction with disiloxane disilanol (**b**) and preparation of cyclolinear PBS from boric acid and diphenyl dichlorosilane (**c**).

It is important to note that, owing to the availability of silicon derivatives with various functionalities (from 1 to 4) and boron compounds (from 1 to 3), PBSs with both linear and branched structures can be obtained. This scheme is convenient in the directed synthesis of high-molecular PBSs. For example, using elementary reaction 2 (Scheme 3), the three-functional boric acid can be used as a branching center to build up the siloxane chains by adding the corresponding dialkyldichlorosilanes (Scheme 4a, b). The structure of the final PBSs depends not only on the functionality of the initial silicon and boron derivatives but also on their stoichiometry. For example, if the ratio of boric acid to diphenyldichlorosilane is changed from 3:1 to 3:2, a cyclic copolymer is obtained (Scheme 4c) in a nearly quantitative yield [59].

**Scheme 5:**
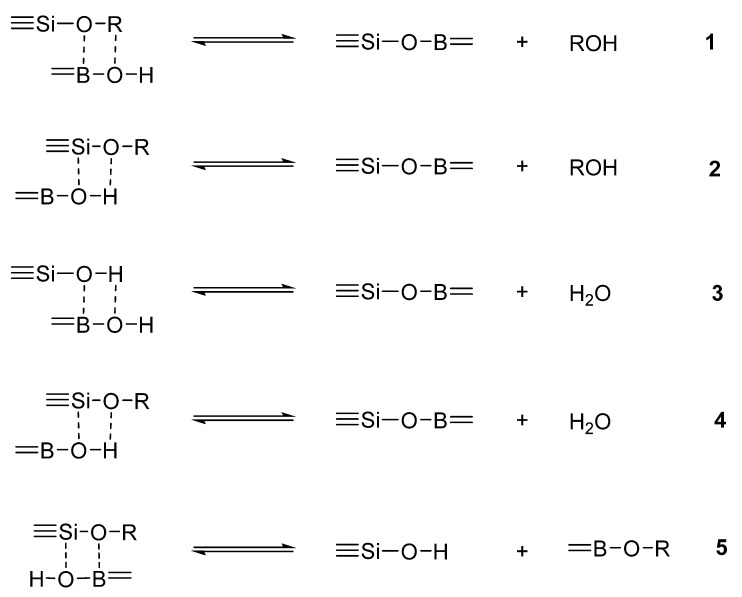
The possible reactions during sol-gel processes.

Furthermore, PBSs with various Si:B ratios were obtained by the reaction of diphenylsilanediol with boric acid in various solvents: dibutyl ether, toluene, and pyrrolidine [60]. Yet, another common method for the synthesis of PBS gels used the sol-gel process involving boronic acid derivatives with alkoxysilanes or silanols by equations of types 3–6 (Scheme 3). The key point of this method lies in the fact that the hydrolysis of organoborates occurs faster than that of alkoxysilanes [61]. It was found by ^11^B and ^29^Si NMR methods that during these processes, the equilibrium of reactions 1–4 (Scheme 5) is shifted toward the formation of the borosiloxane bond at early conversion stages and then shifts in the opposite direction when enough water is formed for hydrolysis.

However, as the resulting gels are dried, the equilibrium shifts toward the formation of the borosiloxane bond. The possibility to shift the equilibrium toward PBS formation, along with the simplicity of reactions and the availability of the starting compounds, make the sol-gel technique of PBS synthesis an attractive and simple method. Nevertheless, as further studies showed, PBS-based materials obtained by the sol-gel method are hydrolytically unstable [62].

**Scheme 6:**
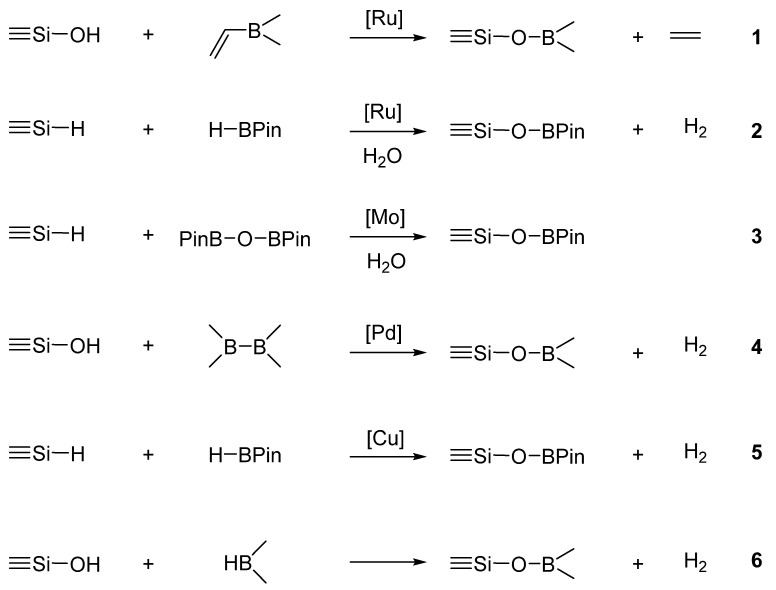
Modern methods used to create the borosiloxane bond.

The high demand for PBSs in various fields provides an impetus to a search for new ways of their synthesis. These approaches often include metal-complex catalysis, modern reactions, and rejection of harmful and toxic starting compounds and side products. For example, over ten years ago, Marciniec’s team developed a synthesis of compounds with the Si-O-B bond (Scheme 6, reaction 1) based on reactions of boron compounds comprising a vinyl function with silanols on ruthenium catalysts [63,64]. It was shown that the conversion of the starting boron compounds approached 90–100%. However, this approach was demonstrated only with boropinacol compounds, the practical utility of which is questionable (pinacol protection is removed under more drastic conditions than the borosiloxane bond undergoes cleavage). A similar approach using ruthenium catalysts (Scheme 6, reaction 2) was suggested in [65]. The difference from previous works lies in the use of hydridosilanes that are oxidized in situ to silanols during the reaction. This approach is justified because hydridosilanes are more readily available.

Yet, another method for the synthesis of boropinacol derivatives is the catalytic reaction of hydridosilanes with bisboryl oxide (PinB-O-BPin) in the presence of molybdenum catalysts (Scheme 6, reaction 3) [66]. The reaction yields approached 90%, but this method may have limited use, i.e., exclusively for the synthesis of boropinacol compounds. In addition, the bisboryl oxide reagent used in the reaction is not commercially available. 

**Figure 4:**
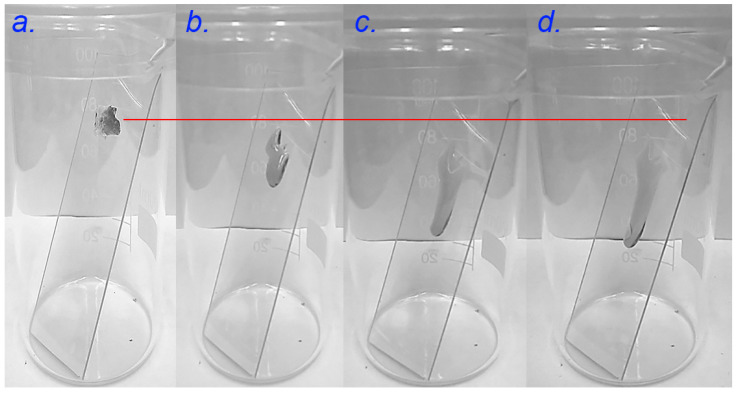
PBS sample placed on an inclined slide glass at different time intervals: after 1 day (**a**), 3 days (**b**), 6 days (**c**), 8 days (**d**).

It was shown in a work titled “A convenient and clean synthetic method for borasiloxanes by Pd-catalysed reaction of silanols with diborons” [67] that borosiloxanes could be synthesized using bis(pinacol diborate) (Scheme 6, reaction 4). It should be noted as a reference that this reagent currently has a market price of about 1000 EUR /100 g. is The work is noteworthy where copper catalysts were used for the synthesis of borosiloxanes (Scheme 6, reaction 5) [68]. An advantage of this work is that it used a certainly promising approach with commercially available catalytic systems. However, as shown by the authors, a difficulty lies in the low conversion provided by the process if it ensures high selectivity. If attempts were performed to increase the conversion, the selectivity of the process toward the formation of the target products changed greatly. In one of their recent works [69], the authors show that the reaction between silanols and hydridoborates can be implemented without catalysts (Scheme 6, reaction 6). Undoubtedly, this work is a breakthrough in the field of creating the borosiloxane bond. However, the starting compounds used for the synthesis strongly limit this approach in terms of scaling and cost reduction. One of the most promising methods of creating Si-O-B bonds was suggested by Rubinsztajn [70] as an analogy to the Piers-Rubinsztajn reaction discovered earlier, which occurred between silylhydride and alkoxysilyl functions and resulted in the formation of a siloxane bond [71,72,73]. It was shown that the reaction of diphenylsilane and trimethylborate in the presence of tris(pentafluorophenyl)boron provided a cross-linked rubber-like material with a conversion close to 100%. The resulting cross-linked polyborosiloxane showed a mass loss of 10% at temperatures above 400 °C. In subsequent studies, cross-linked polyborsiloxanes with various topologies were obtained by cross-linking in the Piers-Rubinsztajn reaction between the terminal hydridosilyl groups or those distributed along a polysiloxane chain, and a di- or trifunctional alkylborate [74]. It was shown in that work that cross-linked polyborosiloxanes were resistant against atmospheric moisture and could remain in a high humidity chamber for 10 days. In addition, it was shown that the obtained PBSs have the same set of rheological properties as the “classical” Silly Putty—elasticity and viscosity. Figure 4a shows that a PBS sample, which originally looks similar to an elastic mass, softens after a few hours and begins to flow under the action of gravity (Figure 4b–d).

Thus, the Piers-Rubinsztajn reaction is among the simplest and most up-to-date methods for generating compounds with Si-O-B bonds and, in particular, cross-linked borosiloxanes resistant to hydrolysis unlike the analogues obtained previously [75].

To summarize, we can state that, historically, the first ways to obtain PBSs utilized simple reactions that provided the desired products using the sol-gel method. However, despite the time that passed since polyborosiloxanes had been synthesized for the first time, the issue of increasing the hydrolytic stability of PBSs has not yet been resolved completely, which has strongly limited their application as functional materials. In recent decades, new approaches to the synthesis of PBSs have been developed, including metal-complex catalysis methods that allow compounds with Si-O-B bonds to be obtained in high yields. Still, not all of the suggested methods are suitable for the directed synthesis of PBSs, since they are limited exclusively to small molecules where the borate function is, in almost all cases, protected by pinacoline protection. A major breakthrough in this direction was provided using the Piers-Rubinsztajn that allows PBSs with various architectures to be obtained from commercially available starting compounds using a catalyst and cross-linked into polymer networks. An undoubtable advantage of this reaction also includes the simple conditions of its implementation, often requiring no solvent. However, the main advantage lies in the high hydrolytic stability of the PBS obtained by this reaction.

## 4. Organic PBS Derivatives with Phenylborate Groups

**Figure 5:**
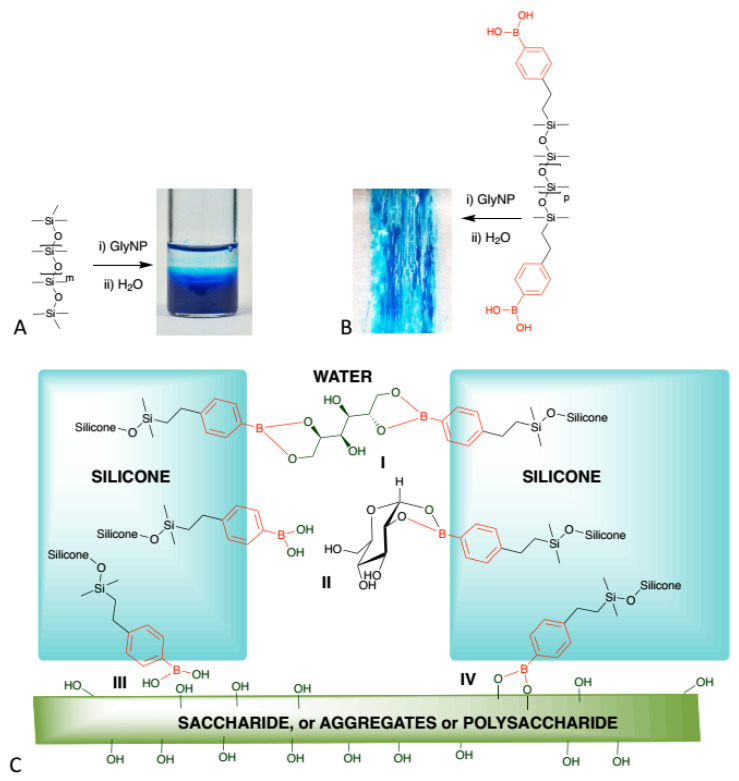
Synthesis of silicone elastomers by cross-linking of phenylborate groups in organic PBSs with saccharides: addition of an ordinary siloxane with phytoglycogen and 35 nm spherical monodisperse nanoparticles (GlyNP) to water (A); addition of siloxanes with phenylborate terminal groups results in a dispersion (B); formation of elastomers with sorbitol (C). Reprinted with permission from [78]. Copyright 2022 Royal Society of Chemistry.

Apart from PBSs, the synthesis of materials whose molecules do not contain Si-O-B bonds as such but that can be considered as polysiloxanes with borate functional groups arranged outside the main chain is actively developing. It is known that borate groups can form labile intermolecular hydrogen bonds. Thus, by incorporating borate functions into the polymer chain it is possible to create an easily degradable network and hence change the physical characteristics of materials based on them [76]. This approach became popular for creating various smart materials that change properties under the impact of various external factors [77]. Moreover, siloxanes with a borate function can be used as an alternative to classical two-component systems that are cross-linked by catalysts based on transition metals. Polysaccharides, renewable natural raw materials that form labile hydrogen bonds with borate groups of polysiloxanes due to a large number of alcohol groups, are used to cross-link the chains. At the same time, polysaccharides also act as a filler. In fact, it was shown [78] that on the addition of an ordinary siloxane with phytoglycogen and 35 nm spherical monodisperse nanoparticles (GlyNP) to water, phase separation occurred (Figure 5A). However, the use of siloxanes with phenylborate terminal groups results in a dispersion (Figure 5B) due to the interaction of phenylborate groups with the alcohol groups of the phytoglycogen. If sorbitol is used, the binding constant of phenylborate groups increases, which results in the formation of elastomers (Figure 5C).

**Figure 6:**
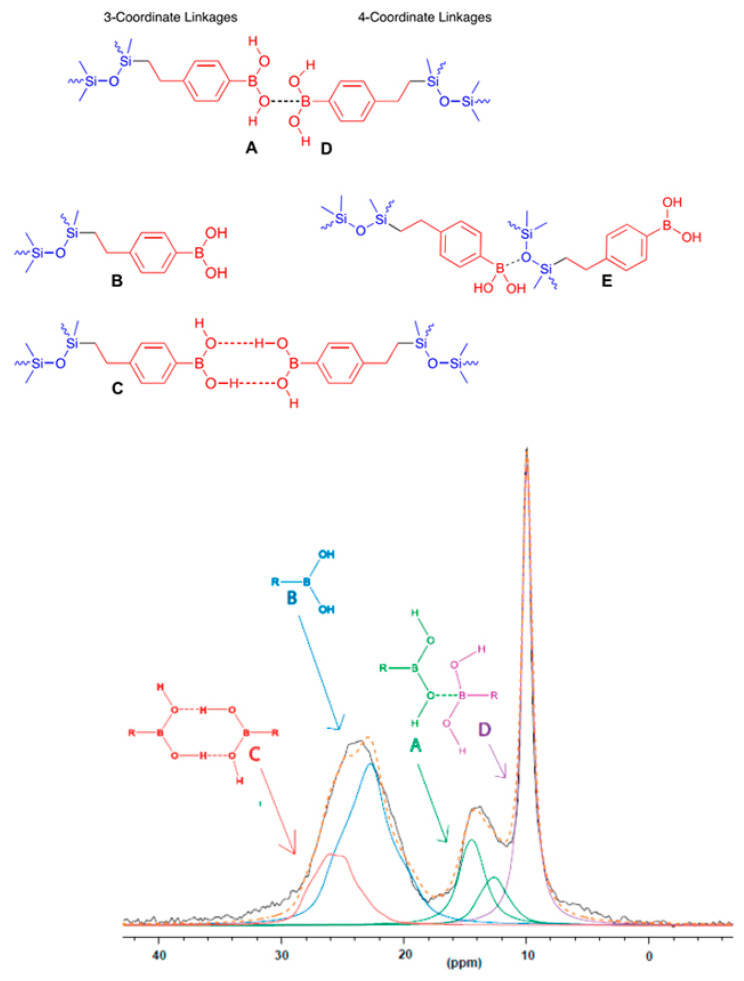
Types of noncovalent interactions between phenylborate moieties in organic PBSs and the type of MQMAS NMR spectrum that confirms their presence. Dative bonding between boronic acids (A), free boronic acid (B), and hydrogen-bonded boronic acids (C). Four-coordinate: dative bonding between a boronic acids (D) and dative bonding between a boronic acid and oxygen on the PDMS backbone (E). Reprinted with permission from [79]. Copyright 2019 American Chemical Society.

It was noted that incorporation of borate groups into polysiloxanes led to their self-association in aqueous solutions and increased biocompatibility [79]. In this work, all types of interactions that exist in solutions of siloxanes with phenylborate groups were systematized. The assumed forms of interactions were confirmed using MQMAS NMR (Figure 6). The following interaction types were shown to be possible: (A) dative bonding between boronic acids, (B) free boronic acid, and (C) hydrogen-bonded boronic acids. These four-coordinates are also possible: (D) dative bonding between a boronic acids and (E) dative bonding between a boronic acid and oxygen on the PDMS backbone.

Siloxanes with protected phenylborate groups demonstrate an interesting effect. Under ordinary conditions, these polymers are viscous liquids similar to polydimethylsiloxane. However, the protective groups are easily hydrolyzed in the presence of moisture and, at the same time, interchain interactions occur, which results in a change in the physical characteristics of the polymer system: it becomes an elastic film with a Young modulus of 150–170 kPa [79]. The incorporation of electron-donor compounds into the system makes it possible to inhibit the formation of hydrogen bonds between the borate groups and, thus, it can be used to control the rigidity of the networks formed [80].

It is known that phenylboric acids are liable to trimerization that provides boroxines, i.e., six-membered rings with alternating boron and oxygen atoms. The chemistry of boroxines is well studied, including their use for the synthesis of supramolecular structures and materials with the self-healing effect [81,82,83,84,85,86,87].

**Figure 7:**
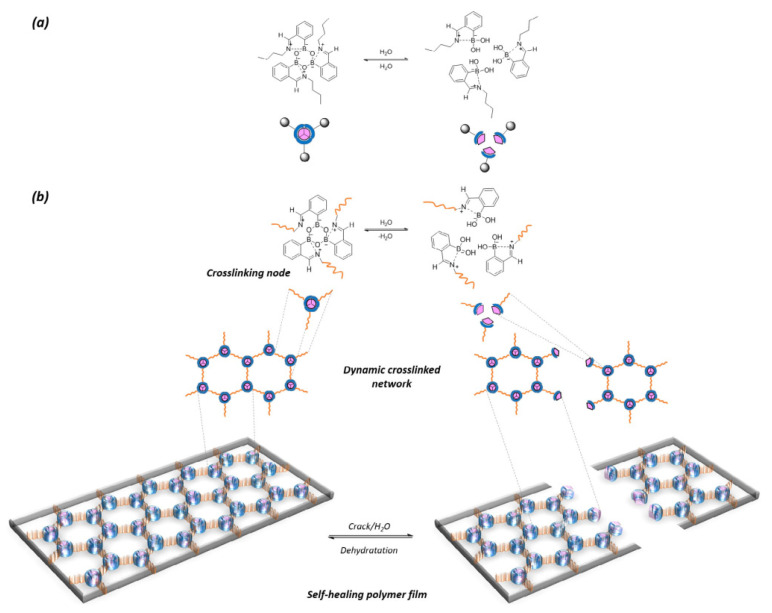
Equilibrium formation of the boroxine ring (**a**) and formation of self-healing films due to dynamically formed boroxine rings (**b**) Reprinted with permission from [8479]. Copyright 1999-2022 John Wiley & Sons, Inc.

**Figure 8:**
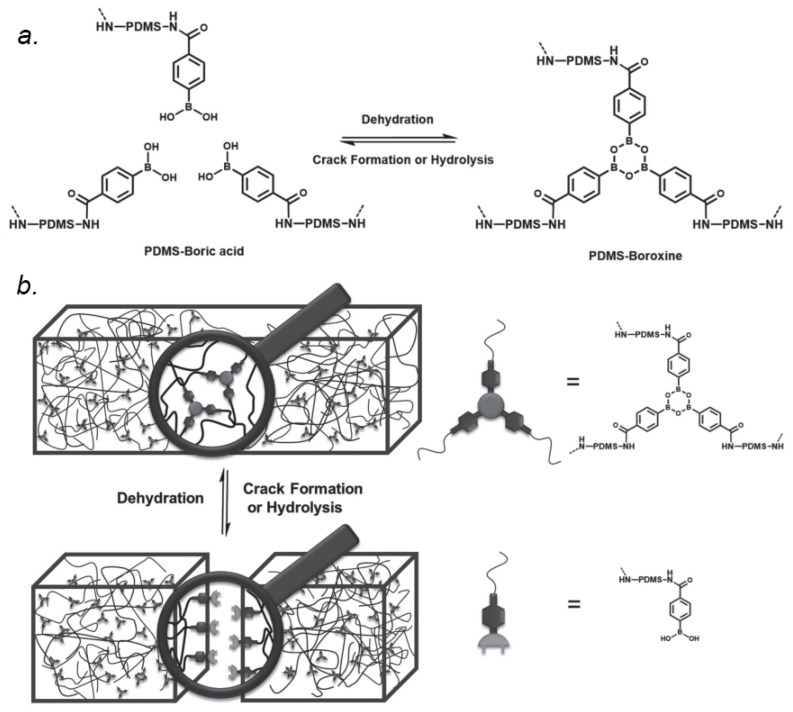
The structure and dynamic process of PDMS-Boroxine (**a**) and the mechanism for the water-enabled healing process (**b**) Reprinted with permission from [7679]. Copyright 1999-2022 John Wiley & Sons, Inc.

The boroxine ring is formed from three equivalents of phenylboric acids with water removal and is degraded upon exposure to air moisture. Figure 7a and Figure 8a show the reversible processes of boroxine ring formation and destruction from three phenylborate residues at the ends of PDMS. Only the spacers used to bind the phenylborate groups to the PDMS chain differ. Figure 7b and Figure 8b clearly demonstrate the self-healing in crosslinked silicones that occurs through dynamic processes of boroxine formation. This effect is used for the self-healing and controlled degradation of polymer networks, including those based on polysiloxanes [45].

## 5. General Approaches to the Synthesis and Further Modification of Organic PBS Derivatives with Phenylborate Groups

The general approaches to the synthesis of PBS derivatives with phenylborate groups are shown in Scheme 5. The concept of this synthesis is based on the use of 1,4-substituted phenylene derivatives with different functional groups. One of the latter is substituted with a boric acid residue using classical organometallic chemistry approaches, i.e., through the intermediate formation of organolithium or organomagnesium compounds, followed by substitution with a boric acid ester. The phenylborate moiety is then protected by a reaction with 1,2-diols, such as ethylene glycol, pinacol, tartaric acid, etc. It was found that if a 1,2-ethylene glycol with electron-acceptor groups, such as tartaric acid, is used to create protective groups, the hydrolytic stability of protected phenylborate moieties decreases abruptly [88]. Brook [73,78,79,80,89] used styrene organoboron modifiers obtained from 4-bromostyrene according to Scheme 5a. The double bond is required here for a simple functionalization of hydrido-containing siloxanes or polysiloxanes using the hydrosilylation reaction. Tartaric acid (R_1_ = COOH, R_2_ = H) is used to protect the borate function. The fact that PDMS with phenylborate groups protected with cyclic tartaric acid ester is readily hydrolyzed with water was used in this case.

**Scheme 7:**
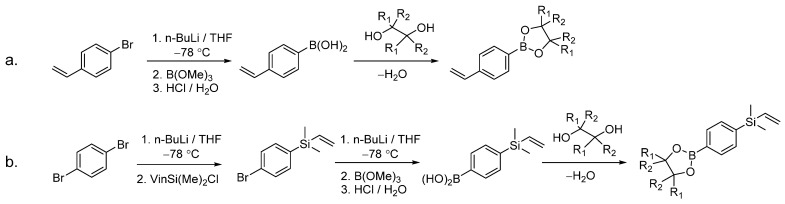
Methods for synthesizing phenylborate modifiers to obtain organic PBSs with phenylborate groups using from 4-bromostyrene (**a**) and 1,4-dibromobenzene (**b**).

However, styrene modifiers have two important drawbacks: they are liable to polymerization even in storage for a long time and the source compounds for their synthesis are quite expensive. Therefore, it was suggested to use a dimethyl vinyl silyl derivative instead of a styrene derivative [90] (Scheme 7b). The synthesis methods are similar but the starting compound, 1,4-dibromobenzene, is commercially available. At the stage of further PDMS functionalization and hydrolysis of the protective group, optimal conditions for its removal were found. In fact, it was shown that if pinacol (R_1_, R_2_ = Me) was used as a protective group, the protective group in the modified PDMS was not removed on stirring in aqueous 1N HCl solution or refluxing in aqueous sodium carbonate solution. However, if ethylene glycol was used (R_1_, R_2_ = H), the protective groups were readily hydrolyzed with water or on passing PDMS solutions with protected phenylborate groups through a silica gel layer. The only drawback of the suggested synthetic scheme is that two stages are needed to prepare the organolithium derivatives. However, they can be replaced with organomagnesium derivatives, which would significantly reduce the cost and complexity of the synthesis.

Apart from the two schemes for synthesizing phenylborate modifiers considered above, other methods to this end also exist. For example, in the example already discussed above [84] a phenylborate modifier with a carboxyl group at position 4 is used to functionalize PDMS with terminal amino groups using the amidization reaction.

Thus, the general methodology for the synthesis of phenylborate modifiers involves the use of 1,4-substituted aromatic compounds with different functional groups. Most often, a halide bound to an aromatic ring is replaced with a lithium derivative, which is then converted to a borate. The second functional group is used in the reaction with PDMS.

Polysiloxanes with phenylborate groups are of interest not only in terms of materials science but also in terms of chemistry. The phenylborate moiety has a great synthetic potential and can be used to obtain a wide range of compounds. For example, the Suzuki-Miyaura [91], Petasis [92,93], and Chan-Lam reactions [94] can be performed on the basis of phenylboric acids.

**Scheme 8:**
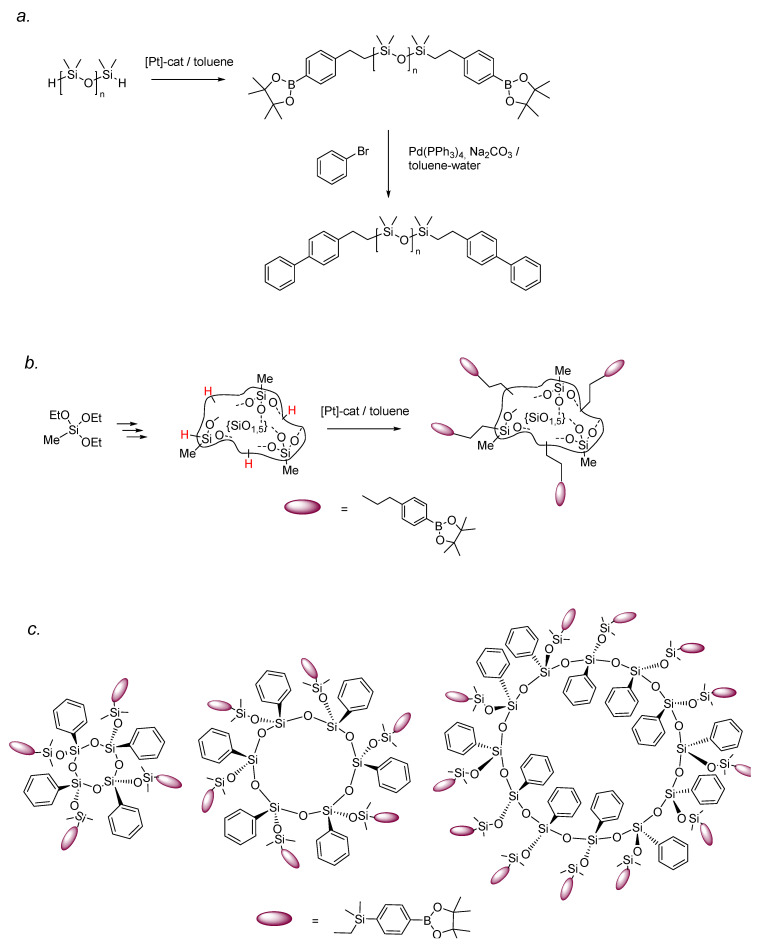
Use of organic PBSs with phenylborate groups in the Suzuki reaction (**a**), synthesis of silsesquioxane nanogels modified with phenylborate groups (**b**) and cyclosiloxanes with phenylborate moieties (**c**).

It has been shown that linear and cyclic siloxanes with protected phenylborate groups can be obtained using simple synthetic methods. Moreover, despite the low stability of polysiloxanes in alkaline media, it is possible to perform the Suzuki reaction with a complete conversion without destroying the siloxane bonds (Scheme 8a) [90,95]. The functionalization of silsesquioxane nanogel surfaces with phenylborate functions for further modification by various organic substrates appears to be a promising approach (Scheme 8b) [96]. Cyclosiloxanes with a various number of links were obtained and modified with phenylborate groups (Scheme 8c).

## 6. Conclusions

In this review, two groups of compounds that can be called by the general term “borosiloxanes” were considered. The first group includes compounds containing the Si-O-B bond. The second group includes siloxanes with phenylborate groups. They can be presented using the general formula ≡Si-Ar-B(OH)_2_. Despite the absence of the Si-O-B bond as such, the behavior of these molecules is similar to that of the “real” borosiloxanes, which is obviously due to the similar nature of intermolecular interactions in these systems.

As shown in this brief review, the field of PBS applications is very wide and continues to expand and may form a separate field of organoelement polymers in the future. This is primarily due to the unique mechanical and rheological properties of PBSs that allow for the use of them as active armor, as artificial leather, for creating soft robots, and in sensors. On the other hand, they have occupied a niche in the production of fire-resistant ceramics and flame retardants due to the high thermal stability of PBSs. As for the second group, its application field is related to the self-association of phenylborate groups that is used to reach the self-healing effect. Moreover, the presence of phenylborate groups is of considerable interest in terms of the preparation of functional materials by various reactions involving these groups.

To summarize, we can predict with certainty that the future of PBSs lies in the expansion of their use as a new class of functional materials and in various kinds of protective coatings. It is obvious that improved methods for their production leading to an increase in their stability favor the promotion of PBSs in various high-end areas of modern technologies.

## Data Availability

The data presented in this study are available on request from the corresponding author.
